# Hearing the physical condition: The relationship between sexually dimorphic vocal traits and underlying physiology

**DOI:** 10.3389/fpsyg.2022.983688

**Published:** 2022-11-03

**Authors:** Shitao Chen, Chengyang Han, Shuai Wang, Xuanwen Liu, Bin Wang, Ran Wei, Xue Lei

**Affiliations:** ^1^Department of Psychology, College of Education, Hangzhou Normal University, Hangzhou, Zhejiang, China; ^2^School of Psychology, Shenzhen University, Shenzhen, Guangdong, China; ^3^School of Business Administration, Zhejiang University of Finance and Economics, Hangzhou, Hangzhou, China

**Keywords:** voice, pitch, formant frequencies, sexual dimorphism, sex hormone, body size, strength

## Abstract

A growing amount of research has shown associations between sexually dimorphic vocal traits and physiological conditions related to reproductive advantage. This paper presented a review of the literature on the relationship between sexually dimorphic vocal traits and sex hormones, body size, and physique. Those physiological conditions are important in reproductive success and mate selection. Regarding sex hormones, there are associations between sex-specific hormones and sexually dimorphic vocal traits; about body size, formant frequencies are more reliable predictors of human body size than pitch/fundamental frequency; with regard to the physique, there is a possible but still controversial association between human voice and strength and combat power, while pitch is more often used as a signal of aggressive intent in conflict. Future research should consider demographic, cross-cultural, cognitive interaction, and emotional motivation influences, in order to more accurately assess the relationship between voice and physiology. Moreover, neurological studies were recommended to gain a deeper understanding of the evolutionary origins and adaptive functions of voice modulation.

## Introduction

Research has shown that a high degree of consistency in the way people judge other people’s voices (Pisanski and Bryant, 2019). For example, low male voice pitch and high female voice pitch are generally considered more attractive (Skrinda et al., 2014). Evolutionary psychologists have suggested that the consistency in the evaluation of voice characteristics is likely due to the fact that different voice characteristics imply corresponding biological information (e.g., reproductive health and physical fitness), which is highly correlated with corresponding social judgment (e.g., sexual attractiveness, resource appropriation; Feinberg, 2008; Puts et al., 2012). Previous studies have often linked human sexually dimorphic vocal traits to sex hormones, body size, and physique, and this paper firstly introduced the widely studied sexually dimorphic acoustic parameters of voice, and then collated and synthesized a review of research on the human sexually dimorphic vocal traits and these three physical signs.

## Overview of the human voice

### Voice related parameters and measurements

The famous Source-Filter Theory was developed by Fant in the 1960s to describe how humans and most mammals produce sound (Fant, 1960). Sound production can be divided into two parts, the source and the filter. Specifically, the source is generated by the vibration of the vocal cords, which are vibrated by the air exiting the lungs and passing through the windpipe, and then impacting the vocal cords, causing them to vibrate and generate the Fundamental frequency (F0). The filter refers to the supraglottal vocal tract, which are spaces that shaped by pharynx, soft palate, tongue, oral cavity, nasal cavity, and sinuses. The supraglottal vocal tract changes its structure (position of the soft palate, tongue) to change the length and size of the vocal tract transiently, thus changing the frequency of the resonances generated by sound reflections in the vocal tract. This process generates resonant frequencies, and the peak values are called Formant frequencies (Titze and Martin, 1998). Finally, by superimposing the source sound with the resonances of sound reflection in the vocal tract, humans and most mammals eventually achieve vocalization. It is as if the tune of a violin is not only related to the frequency of string vibration (the source), but also the resonances (filters) produced by sound reflection in the cavity of the instrument.

The perceptual component of human vocal fundamental frequency is commonly referred to as voice pitch. Generally, larger vocal folds vibrate at a lower frequency than the smaller ones, resulting in a relatively low F0; however, regardless of volume, F0 increases when the vocal folds are stretched and under tension, so this characteristic is determined by the volume, length and tension of the vocal folds (Titze, 2011; Hollien, 2014). The tone is related to the formant frequencies in the upper larynx. Since formant frequencies include a range of formant frequency values (e.g., first resonance peak frequency F1, second resonance peak F2, etc.), it is often converted into a single value to represent its distribution in research, and there are currently 12 methods (Pisanski et al., 2014). The commonly used of which are: formant disposition (P*f*), estimated vocal track length (VTL), and formant dispersion (D*f*). The relative positions of resonance peaks (especially F1 and F2) play a key role in speech production and perception, and play a greater role than F0 in non-tonal languages^1^ (Titze and Martin, 1998). Longer vocal tracts produce a relatively lower and tighter formant dispersion than shorter ones, while manipulation of the tongue, lips, jaw, and soft palate can also alter the shape of the vocal tract, affecting the relative dispersion of formant frequencies and thus producing different articulations (Pisanski et al., 2014).

The sexual dimorphism of the human voice is currently of great interest in the fields of evolutionary psychology and acoustics.

### Gender dimorphic features of the voice

From the evolutionary psychology perspective, sexual selection and parental investment theories suggest that one sex that invests less in its offspring will generate stronger intra-sexual competition during reproduction (Buss, 2019). In many humanoid primates, males tend to experience stronger intra-sexual competition and possess external traits (e.g., facial and physical traits) that play a role in winning mates (Buss, 2019; Aung and Puts, 2020). It has also been suggested that an animal’s vocalizations may also reflect its formability (Sell et al., 2010), and that low-frequency vocalizations may help males gain mates by intimidating other males and/or attracting females, thus allowing males to develop a lower F0 compared to females (Puts et al., 2014, 2016).

The human voice exhibits significant sexual dimorphism (Puts et al., 2016). In males, there is a significant increase in androgens, thickening of the vocal folds, and increasing of vocal fold length during puberty, resulting in a significant decrease in F0 (Fouquet et al., 2016; Markova et al., 2016). This causes a significant decrease in F0. In adulthood, the length of the vocal folds is about 60% longer and the F0 is about five standard deviations lower in men than in women (Puts et al., 2016). Individuals with low pitch usually have longer vocal folds and less muscle tension on the vocal folds, which vibrate at lower fundamental frequencies (Titze and Martin, 1998). Many studies have found that the lower pitch of male plays a significant role in conveying the impression of dominance to other individuals (Puts et al., 2006; Hodges-Simeon et al., 2011; Hill et al., 2013; Puts, 2016). Puts et al. (2006) speculate that males and females may use the significant gender differences between F0 and resonance peak frequency to convey various gender-related attributes, for example, to convey information related to physical dominance, heterosexual attraction, threat signals (Cartei et al., 2012).

## The human voice and sex hormones

### The male voice and testosterone

The effects of androgens such as testosterone on the voice are mainly pitch-based. In males, testosterone levels rise during puberty, promoting the development of the laryngeal tissues and framework and the formation of laryngeal nodes. At the same time, the muscular and mucosal layers of the vocal folds thicken, the vocal folds become longer and wider, and male pitch typically decreases after puberty (Harries et al., 1997). Harries et al. (1997) recorded data on a group of boys aged 13–14 years, including their speaking pitch and their salivary testosterone levels, over a period of 1 year, during which these boys experienced vocal changes specific to puberty. Unfortunately, this study did not find a correlation between testosterone levels and pitch, but interestingly, testicular volume was associated with changes in pitch, i.e., the larger the testicular volume, the lower the pitch (Harries et al., 1997). Previous studies have suggested that changes in male voice are completed at puberty, and thus there is no reason to think that pitch would be associated with testosterone levels in the internal circulation of individuals after puberty. However, a small number of studies have found that testosterone levels in the internal circulation are associated with acoustic parameters (F0 and P*f*) of the adult male voice. Meuser and Nieschlag (1977) found that tenor singers had lower testosterone/estradiol ratios than baritone and bass singers. In two separate studies, it was found that in young male samples, there was a negative correlation between testosterone levels and pitch (Pedersen et al., 1986; Dabbs and Mallinger, 1999). Later, Bruckert et al. (2006) also found in their study that men with less discrete formant frequencies had higher testosterone levels, but the study did not find a correlation between testosterone levels and pitch.

The reasons for the divergence in these studies may be as follows: firstly, testosterone levels in saliva and serum of adult men vary dynamically throughout the day, with testosterone levels peaking in the morning and reaching their lowest point in the evening, and inconsistency in sampling time points may cause biased experimental results (Campbell et al., 1982). For that matter, given the day-to-day variability of testosterone, researchers should use a more rigorous approach to measuring testosterone levels. For example, Evans et al. (2008) collected saliva samples from subjects at 9 am, 12 noon and 3 pm and explored the relationship between testosterone levels and voice parameters, and the results supported previous findings that there was a negative correlation between testosterone levels and fundamental frequency, and to a greater extent than in previous studies. It is inferred that voice pitch can provide a true signal about an individual’s hormone levels (Evans et al., 2008). In addition, some medical studies suggest that if males do not transition well during the voice change period, this may lead to adolescent falsetto, also known as male to female voice tone, a functional vocal disorder that can be treated with appropriate doses of testosterone to reduce vocal frequency (Zhuang and Liu, 2021).

In sum, part of the studies found a negative correlation between testosterone levels and men’s pitch, and the diurnal shifts of the testosterone levels may cause difficulty getting consistent results, when the test timing is not well controlled.

### The female voice and estrogen

The larynx is an important target organ for sex hormones. For women, the vocal fold mucosa proliferates and increases glandular secretion and capillary permeability in response to estrogen, while progesterone acts on top of estrogen to inhibit estrogen-induced hyperplasia of the vocal fold mucosa and glandular secretion and to reduce capillary permeability (Kirgezen et al., 2017; Kim et al., 2020). During the pubertal phase, women experience a mild thickening and lengthening of the vocal folds and a decrease in pitch of approximately one-third of an octave in response to estrogen and progesterone (Zhuang and Liu, 2021).

It has been shown that the female voice changes cyclically with the menstrual cycle, with the follicular phase being the beginning of the menstrual cycle, a period when estrogen levels are significantly higher while progesterone levels are significantly lower, and that this hormonal change leads to vocal fold edema and allows increased blood flow through the vocal folds, and that polysaccharides in the vocal folds break down more easily and bind water more readily, which in turn further promotes the accumulation of fluid in the vocal folds (Kadakia et al., 2013). In addition, blood vessels in the nasal cavity dilate, thus affecting airflow, and the hormonal environment can also lead to increased reflux symptoms by slowing down gastric motility (Kadakia et al., 2013). During the luteal phase, progesterone levels increase much more than estrogen levels, progesterone promotes the shedding of the laryngeal epithelium and inhibits its proliferation, and it also causes glandular secretions to become more viscous, leading to a decrease in the frequency of vocal cord vibrations (Kadakia et al., 2013). Kadakia et al. (2013) postulated that these changes are the main cause of vocal changes during the female menstrual cycle. In a study that recorded voice audio from female subjects at different times during their menstrual cycle, and then rated the attractiveness of the subjects’ voice audio by 30 men and 30 women, they found that voice attractiveness ratings increased significantly throughout the menstrual cycle as the odds of pregnancy increased (closer to ovulation), suggesting that women’s voices may provide reproductive signals related to sex hormone fluctuations (Pipitone and Gallup, 2008).

During menopause, women’s voices change dramatically as their estrogen and progesterone levels decline. At the beginning of menopause, follicle stimulating hormone (FSH) and luteinizing hormone (LH) remain at a high level and the ovaries continue to produce androgens. For women with high fat reserves, these secreted androgens are converted into estrogen, maintaining the impact of estrogen on the body. However, for some women with low fat reserves, no androgens can be converted, thus leaving androgen levels relatively high, which reduces the pitch of the voice and causes irreversible changes (Strauss et al., 1985).

In sum, due to the impact of hormone change, women’s voice change during puberty, menstrual cycle, and menopause.

Furthermore, data from both women and men suggested that human sex hormone level change can influence individual’s voice, especially pitch. Because the sexually dimorphic vocal traits are impacted by sex hormones, and these hormones are linked with reproductive and health viability in men and women (Venners et al., 2006; Almeida et al., 2017). Therefore, it is possible that the sexually dimorphic vocal traits signal reproductive advantage (Apicella et al., 2007; Atkinson et al., 2012) and then sexual selection favored these sexually dimorphic vocal traits (Puts, 2016), which in turn amplified the sexually dimorphic differences of voice between men and women. The similar phenomenon is also replicated in human voice and other physiology that are important to mate competition, such as men’s body size and physique.

## Voice and body size

Studies on animals have shown that large body size is generally preferred by the opposite sex. For example, female cichlid fish prefer to spawn near larger males because larger males are better able to provide territorial defense as a means of protecting their offspring (Keenleyside et al., 1985). For territorial monogamous species, females also prefer larger males as larger males tend to gain more territory and thus provide better environmental conditions for females to raise their offspring (Eberhard and Ewald, 1994; Nimje et al., 2021). Evidence from animal vocalizations studies has shown that acoustic signals can provide information on physical characteristics, such as body size, age, and sex. The formant dispersion was found to be a reliable predictor of body size in macaques, as measured by radiographs and computer graphics techniques (Fitch, 1997). In a study of domestic dogs, a significant correlation between formant dispersion and body size was found by recording the acoustic signals of domestic dogs growling (Riede and Fitch, 1999).

In human, body size is often an associated with one’s competitiveness, social status, and attractiveness. It is also an important cue for individuals to effectively assess the strength of their competitors and the quality of their mates (Fitch, 2000). It is often assumed that men with low voices are more attractive to the opposite sex and more dominant over the same sex, so what exactly is the relationship between the human voice and its body size?

### Pitch and body size

It has been found that lower F0 in males predicted a number of parameters related to physical signs, such as shoulder circumference and chest circumference as well as height and weight (Evans et al., 2006; Pisanski et al., 2014; Aung and Puts, 2020). Sensory exploitation theories of sexual selection explain this phenomenon as a simple physical property of the world, as if a rock emits lower frequency vibrations when struck with a stick on a larger rock (Titze and Martin, 1994). The perception of a lower male pitch as more dominant simply reflects a response of the organism to objects that emit lower frequency vibrations. There is a clear manifestation of this not just in humans, but throughout the animal kingdom—the perception that bass tones are loud and frightening—suggesting that this sensory-biased response is evolutionarily long-standing (Morton, 1977). One study found that congenitally blind people and sighted people alike perceived that males with lower pitch should be larger, suggesting that visual learning is not required for that auditory perception (Pisanski et al., 2017).

Sensory exploitation theories of sexual selection also suggest that the “lower is louder” heuristic is commonly used in the processing of auditory stimuli. As a result, the perception that bass males have greater size and dominance is likely to be a mere by-product of this heuristic (Rendall et al., 2007). So how does the “lower is louder” heuristic filter out bass males? Feinberg et al. (2018) suggest two possible pathways. First, if all else being equal, bass men exploit women’s sensory bias that “bass is bigger,” causing women to perceive bass men as having a larger size and more dominant position, which leads women to actively choose bass men. Consistent with this possibility, artificially lowering the pitch of men’s voices in the experiment had a positive effect on the opposite sex’s assessment of their attractiveness. Second, all else being equal, men with lower pitches are more likely to win in same-sex competition. Low-pitched men take advantage of other men’s sensory bias that ‘bass is bigger’, causing other men to perceive bass men as larger and more threatening to them, causing other men to be less confident of winning or even to flee the battle, thus making it easier to win intra-sexual competition and giving bass men an evolutionary advantage (Feinberg et al., 2018). Consistent with this possibility, artificially lowering the pitch of male voices in experiments has a positive effect on same-sex assessments of their dominance (Jones et al., 2010).

However, there is also research evidence that the relationship between human voice and true body size is not robust. It has been found that when controlling for sex and age, pitch has a very limited role in predicting body size in many mammals (Fink et al., 2003; Ey et al., 2007). Studies on human have also found that pitch is similarly unsatisfactory in predicting body size in humans (Pisanski et al., 2014). Furthermore, Pisanski et al. (2014) using meta-analysis found that, after controlling for sex, the predictive effect of pitch on body size explained at most 2% of the variance. Furthermore, in studies of adults, both male and female, some research evidence does not support a significant correlation between pitch and body size (González, 2004; Rendall et al., 2005; Evans et al., 2006).

### Formant frequencies and body size

Formant frequencies may provide more clues about body size than pitch. Unlike the vocal folds, the length of the vocal tract is largely limited by the skeletal structures that make it up, the length of the neck and the size of the skull; in turn, these structural features are both determined by, and to some extent determine, body size. Three studies have demonstrated a correlation between formant frequencies and adult height in males (Rendall et al., 2005; Bruckert et al., 2006; Evans et al., 2006), while a similar correlation was found in a study of a female sample (Collins and Missing, 2003). A meta-analysis found that formant frequencies of the human voice explained approximately 10% of its body size information (Pisanski et al., 2014). Furthermore, Pisanski et al. (2014) suggest that, given a sufficient sample size, formant frequencies can explain variations in female body size and that women’s voices may carry information about their waist-to-hip ratio (Pisanski et al., 2016b). This finding is consistent with the growing literature that the ‘hourglass’ shape of a woman’s body is a key indicator of her age, fertility and health status (Singh and Singh, 2011; Pisanski and Feinberg, 2013), so a beautiful female voice may suggest a reproductive advantage.

Unlike the sensory exploitation theories of sexual selection, Aung and Puts (2020) suggested that the long-standing tendency to associate low-frequency vocalizations with larger body size in vertebrates may have a role in assessing body size among and within species in natural competition (Morton, 1977). Furthermore, in response to the question of whether organisms can be deceived by volitional vocalizations used to exaggerate body size, game theory models theorize that such deceptive signals must be rare in order for the signal system to remain evolutionarily stable (Grafen, 1990). Otherwise, the following two outcomes may occur: either the organism evolves to ignore the signal altogether; or the organism evolves to be able to distinguish deceptive signals from real signals that provide accurate physiological signs (Garcia and Ramirez, 2005; Pisanski and Reby, 2021). In studies on humans, it has been found that men with lower voices tend to earn more, win more political elections, have more sexual partners and leave more offspring (Apicella et al., 2007; Mayew et al., 2013; Klofstad, 2016). If the male voice was unrelated to physical signs, evolutionary direction should have predisposed one to ignore this signal. Then, why is it that men with lower voices are perceived to be more attractive and dominant, and in the real world, they are more successful? The most likely answer is that the voice signal is, at least in part, accurate and true (Puts et al., 2019). One recent work also provides strong evidence that some features of male voices are related to their physiology (albeit not perfectly; Pisanski and Reby, 2021).

## Human voice and physique

Due to the potentially costly nature of intra-sexual conflict, individuals may prefer to reduce costly conflict by predicting each other’s physique, such as strength, fighting ability, and even social status (in the form of dominance), through non-combative approaches (e.g., appearance, voice) (Andersson, 1994). The relationship between sound and fighting ability has been studied in animals from early on. Several studies have shown that in many terrestrial mammals, such as giant pandas, sea lions, horse, deer and domestic dogs, acoustic signals can be used to determine each other’s relative position in aggressive vocalizations, especially in male competition (Reby et al., 2005; Charlton et al., 2010; Taylor et al., 2010; Charrier et al., 2011; Pitcher et al., 2015). These acoustic signals not only predict aggression toward each other, but also elicit a fight or flight response from signal receivers based on their relative combat prowess toward each other (Tibbetts and Dale, 2004; Osiejuk et al., 2007; Anderson et al., 2012). It follows that acoustic cues may contain information about individual physicality that is relevant to individual conflict, particularly intra-sexual competition.

Studies on humans, it has also been shown that there may be a correlation between their voice and strength and combat power. In a cross-cultural study, researchers found that in an American sample, P*f* (formant position, a formant frequencies calculation) predicted individual upper limb strength, while F0-SD (standard deviation of F0) predicted self-reported physical aggression and was slightly negatively correlated with arm strength (Puts et al., 2012). In addition, F0 declines sharply with male puberty and shows a high degree of gender dimorphism in adulthood, thus also providing information about variables such as strength. For example, F0 explains over 60% of the variance in grip strength in a mixed-sex sample of US adult college students, as well as over 70% of the variance in upper limb strength in a sample of Bolivian adolescent males (Aung and Puts, 2020). Sell et al. (2010) recruited listeners to assess the upper limb strength of voice providers from a sample of eight from four different language groups. The results found that people could assess each other’s strength more consistently, that their judgments were accurate whether assessing familiar or unfamiliar languages, and that they were more accurate in assessing males than females (Sell et al., 2010). In addition, Raine et al. (2018) showed that listeners were also able to judge the relative strength and height of subjects based on their own strength and height by assessing their aggressive language or threatening rants; for example, when assessing threatening rants, male listeners were able to accurately identify subjects who were taller and stronger than themselves in 88% of the experiments, but unfortunately this study did not examine the correlation between strength and acoustic parameters correlation (Raine et al., 2018).

Some researchers have also argued that there is still insufficient evidence to suggest a significant negative correlation between pitch and upper limb strength in men, and that previously reported correlations between pitch and upper limb strength would not be significant when corrected for multiple comparisons (Feinberg et al., 2018). The possible reason for some of the contradictory results of previous studies is largely due to the fact that these studies differed significantly in their measurements, including the measurement of strength, and that upper limb strength or grip strength is only a representation of strength in a local area, or arguably part of the many component modules of strength. Therefore, assessing strength with more precise measurements or by combining multiple measurements is likely to result in a stronger association with acoustic parameters.

Some research has recently begun to focus on the relationship between the human voice and signals of aggressive intent, with Zhang et al. (2021) showing in an experimental study that, at least for males, their lowered pitch served primarily as a signal of aggressive intent, independent of an assessment of their own combat strength (Zhang et al., 2021). This study suggested that although listeners have the ability to judge the strength or combat power of subjects from their voices, the correlation between human voice and strength and combat power remains largely unknown after excluding some invalid or inconsistent findings (Sell et al., 2010; Puts et al., 2012; Hodges-Simeon et al., 2014; Smith et al., 2017; Han et al., 2018; Kordsmeyer et al., 2018).

Taken together, these studies raise the following questions: first, as there is an evolutionary commonality in the structure and function of other mammalian vocalizations and human spontaneous vocalizations, such as laughter (Ross et al., 2009, 2010; Bryant and Aktipis, 2014; Pisanski et al., 2016a, 2022) and infant screams of pain (Lingle et al., 2012; Lingle and Riede, 2014), does human spontaneous vocalization convey information about physiological aspects (e.g., strength) more effectively than volitional vocalization (speech)? Recent works tested this on perceptual level, which found roar-like vocalizations increase the perceived physical strength of vocalizers relative to screams, distressed speech, and neutral speech (Raine et al., 2019; Kleisner et al., 2021). Future research can test this on real physical condition (i.e., the effectiveness of prediction from spontaneous vocalization and speech to real physical strength). Secondly, volitional vocalizations are more complex and diverse in humans compared to other mammals. For example, humans can exaggerate their physiological advantages by using words that exaggerate their strength or physical qualities to influence listeners in judging their physiological indicators, while the content of language also limits human non-verbal vocalization relatively more. Therefore, if interference parameters such as language content, motivational state, and emotional information are further controlled, will acoustic parameters convey physiological information more effectively than previous studies? Again, this question will need to be explored in future experiments. One recent paper reviewed the literature on human nonverbal vocalization, which also introduced new techniques that can manipulate voice (Pisanski et al., 2022). This facilitates future work in controlling confounding variables.

## Research discussion

In summary, there is a correlation between human voice characteristics and physiological signs. In terms of body size, formant frequencies are more reliable predictor of human body size than pitch; in terms of physique, the relationship between human voice and strength and combat power may be correlated, but remains controversial; and in terms of sex hormones, sex hormone levels affect human voice variation and perception of voice. While these studies have provided further insight into the human voice and physiological signs, some conclusions remain divergent and need to be further expanded, deepened and refined. In addition, it is likely that the more consistent social evaluation of voice may also suggest that it is based on certain biological characteristics, for example, a low voice is often perceived as coming from a tall and powerful person, and therefore is easily perceived as a high dominant voice (Banai et al., 2017; Han et al., 2021). It is expected that the study of the social and physiological correspondence between the social evaluation of the voice will receive increasing attention.

At the same time, voice belongs to a comprehensive and multidisciplinary nature, including multiple disciplines such as biology (anatomy, physiology, neurology), psychology (cognitive, developmental, cross-cultural, experimental, social), ethology (including primatology), anthropology, bioacoustics, communication and linguistics. Although there has been considerable interest and a gradual rise in research by researchers from different disciplines on key topics such as the physiological mechanisms of voice control and modulation, the culture and its environmental factors affecting voice modulation, the evolutionary origins and adaptive functions of voice modulation, and the social functions of voice modulation. However, to date, most of the research and publications have been scattered in specialist journals on a variety of topics, and there is a lack of cross-disciplinary cross-dialog, as well as compilation of specialized fields. Recently, the Royal Society Publishing had a special issue that incorporated multidiscipline on the topic of “Voice modulation: from origin and mechanism to social impact” (Leongómez et al., 2021). In the future, interdisciplinary collaboration could be used to increase the avenues of dialog to bridge the blind spots between disciplines, allowing researchers from different disciplines to transcend traditional disciplinary boundaries and thus laying the groundwork for a lasting, interdisciplinary foundation in the field of voice.

In addition, there are a number of aspects of current voice-related research that could be further strengthened.

### The need to control demographic factors

Through voice training, actors and voice imitators can significantly increase or decrease the acoustic parameters of their voices (F0 and formant frequencies; Kreiman and Sidtis, 2011). For example, the political figure—Margaret Thatcher—underwent a long period of voice coaching training to reduce the frequency of her articulation in order to present a more authoritative, leadership image (Karpf, 2006). A growing body of research suggests that people who have consciously trained their voices often spontaneously modulate their voices in everyday communication situations (e.g., dating and job interviews) as a way to lead socially (Pisanski et al., 2016a). In addition, social factors including culture and gender are also important; Cartei et al. (2022) found in a voice imitation task with boys and girls (aged 8–10 years) that children spontaneously masculinized or feminized their voices by lowering or raising their pitch, depending on whether the person they were talking to was typically male (rugby) or female (ballet), suggesting that volitional of voice modulation may emerge early in childhood development. Therefore, the variation of an individual’s own voice across occupations, ages, and ecological contexts, and whether the perceived voice differs from that of the general population, needs to be further explored.

### The need to study culturally diverse groups of subjects

Most of the subjects studied in the past have been Westerners, but listeners in different ethnic and cultural contexts may have different perceptions of the attractiveness and dominance of the voice. At the same time, the voice carries important ‘dynamic’ information, such as regional (accent) or ethnic-specific articulation patterns, which allow listeners to identify physical and psychological characteristics (e.g., trustworthiness) more accurately from the voice (Kreiman, 1997). In addition, voices contain emotional information (e.g., anger and sadness), and groups from different backgrounds may have different emotional recognition of voices. In a cross-cultural study of laughter perception, Kamiloğlu et al. (2022) compared data from Dutch and Japanese listeners and found that listeners from both cultures perceived spontaneous laughter as more positive than volitional laughter. Moreover, listeners could identify whether laughter was produced by speakers from their own culture, suggesting that non-verbal information in human voice can encode cultural identity (Kamiloğlu et al., 2022). Future studies are recommended to examine individuals from different cultures (especially, between tonal and non-tonal languages) to investigate how cultural differences affect voice perception.

### Need to improve experimental design

In previous studies, most experiments have manipulated one acoustic parameter at a time, and the interaction of different acoustic parameters in forming cognitive judgments has not been well documented for listeners (Schild et al., 2020). Previous studies, which have focused on linear relationships, therefore remain largely unclear as to whether acoustic parameters have a curvilinear effect on perception (Puts et al., 2012). At the same time, experimental design makes it difficult to avoid experimenter effects and Hawthorne effects, and linguistic content and motivation can unconsciously influence non-verbal factors. Future research should therefore examine the interaction of different acoustic parameters in natural language and elucidate the relationship between them; and use more contextualized language content in experiments to accurately examine the influence of non-verbal factors.

### Possible effects of verbal emotional messages and motivational states

People are able to convey emotional information in verbal communication, and emotional information usually has positive or negative attributes. At the same time, changes in verbal emotional information are often constituted by direct changes in acoustic parameters such as pitch, formant frequencies and volume (Zheng et al., 2017). Furthermore, subjects in different motivational states during verbal communication may also convey different acoustic parameters; for example, the voice may show different properties in a motivational state of mate choice and in a motivational state of self-preservation (Puts, 2006). Pinheiro et al. (2021) studied two types of non-verbal vocalizations: crying and laughing, and designed an experiment to test whether their volitional and spontaneous vocalizations affect listeners’ perceptions of the speaker’s. The results showed that listeners were able to discriminate between spontaneous and volitional vocalizations and that spontaneous vocalizations were considered more trustworthy than volitional vocalizations (Pinheiro et al., 2021). This partly explains why some earlier studies have not yet found a consistent correlation between acoustic parameters and physiological signs, most likely because the correlation between acoustic parameters and physiological signs would serve emotional and motivational states to some extent. Future studies should carefully differentiate and even investigate whether non-verbal vocalizations (martial arts vocalizations) more reliably convey physiological cues.

### The need for research at the neural level

Many species, including humans, possess the ability to perceive vocalizations. Human infants do not speak or understand language, but they are able to recognize sounds. It was found that in experiments measuring heart rate changes in newborns when they heard different sounds that they had the ability to recognize sounds and identify the voices of their parents (DeCasper and Fifer, 1980). This suggests that the infant’s ability to perceive vocalizations is likely to be acquired before fetal birth (Kisilevsky et al., 2003). Recent neuroimaging findings also suggest that neurocognitive models of voice perception are largely similar to those of face perception, and that different types of voice information can be processed in partially separated functional pathways (Belin et al., 2004). In an experiment on imitating voices, Waters et al. (2021) simultaneously observed and recorded voice anatomy and brain function in trained singers and non-singer controls. In a real-time map of changes, they found that singers were able to adjust their speech more accurately in a task that imitated volume level and pitch, and showed stronger laryngeal neural associations within the right dorsal somatosensory cortex region, suggesting a common neural basis for enhanced vocal control in speech and song (Waters et al., 2021). Future research should examine the effects of perceived vocalizations on neural activity in conjunction with advanced brain imaging techniques to provide a neural-level explanation for listeners’ cognitive judgments of voice.

## Author contributions

SC, XLei, and CH contributed to the conception of the paper. SC and CH wrote the first draft of the manuscript. SW, XLiu, BW, and RW contributed to the manuscript revision, read, and approved the submitted version. All authors contributed to the article and approved the submitted version.

## Conflict of interest

The authors declare that the research was conducted in the absence of any commercial or financial relationships that could be construed as a potential conflict of interest.

## Publisher’s note

All claims expressed in this article are solely those of the authors and do not necessarily represent those of their affiliated organizations, or those of the publisher, the editors and the reviewers. Any product that may be evaluated in this article, or claim that may be made by its manufacturer, is not guaranteed or endorsed by the publisher.

## Footnotes

^1^ Non-tonal languages: Languages that do not use pitch in their utterances to distinguish between words and grammatical meaning, such as English, French, German, etc.
